# The John Sealy Training School—Galveston

**Published:** 1892-07

**Authors:** 


For Daniel's Texas Medical Journal.

### THE JOHN SEALY TRAINING SCHOOL—GALVESTON.

BY "ADNE."

THIS important department of the John Sealy Hospital was organized March 10, 1890, through the untiring efforts of Mrs. Chas. League, aided by numerous benevolent ladies and gentlemen of Galveston.

On March 10th, 1892, six (6) competent and well trained nurses were awarded certificates of proficiency, after two years' actnal experience and actual service in the wards of John Sealy Hospital and nursing the sick in private families.

Having successfully passed through its experimental stage, this institution under the guidance of Miss Dorothea Fick, Superintendent, and Miss Wygant, assistant (both graduates of the Mt. Sinai Hospital Training School for Nurses, New York), already bids fair to rival the training schools of larger cities in the North.

Launched into public favor under such difficulties, this training school deservedly expects recognition at the hands of the medical profession of this State, and should receive financial aid from all wealthy citizens philanthropically inclined. Being an institution of the profoundest charitable nature, the happy influence of which will benefit those living in the remotest parts of our great and rapidly growing State, as well as the citizens of Galveston, the recognition and support of this training school should be general.

Not being especially endowed, the school is dependent for support:

First, by membership fees paid annually to the society.

Second, by life membership.

Third, by scholarship or donation.

The Superintendent will gladly furnish the annual report to those especially interested.

This is not a local institution. Any young woman complying with the requirements of admission is eligible.

Educated, refined healthy young women are given an opportunity to fit themselves for a useful career and provided with means of procuring a comfortable and respectable livelihood.

Applicants are received at any time during the year.

The requirements of admission are :

1. The candidate should, preferably be between the ages of twenty and twenty-five.

2. Must furnish testimonials of good moral character and good health, signed by a physician.

3. Must pass satisfactory examinations in reading, penmanship, simple arithmetic and English.

4. If satisfactorily passed on above requirements, must undergo one month's probation to determine fitness for work, at the end of which time she is retained or dismissed, at the discretion of the superintendent.

During the month's probation the pupil is boarded at the expense of the institution, but receives no remuneration.

The successful candidates become pupil nurses, after signing an agreement to remain two years and obey the rules of the school and hospital.

The pupils reside in the school building, and serve the first year in the wards of the hospital, the second year they are required to perform any duty assigned them by the superintendent, either to act as nurses in the hospital or among private cases of the rich or poor.

During the entire course, the sum of seven dollars ($7.00) per month is allowed each pupil, for dress, text books and other personal expenses, and in no wise is intended as wages, the educational advantages given being considered, a fair equivalent for the services rendered. On completion of two full terms, and on passing satisfactory examination, the pupil nurse receives a diploma and is at liberty to choose a field of labor.

Every opportunity is given the pupil to become thoroughly competent. Thus is afforded, to a class of conscientious and self-sacrificing women aspiring to a higher vocation than heretofore placed within their reach, an opportunity to benefit mankind, and earn a respectable livelihood.

